# Associated factors with health-compromising behaviors among patients treated for oral cancer

**DOI:** 10.4317/medoral.22655

**Published:** 2018-12-24

**Authors:** Rocío Barrios-Rodríguez, José-Antonio Gil-Montoya, Javier Montero, Eva-María Rosel, Manuel Bravo

**Affiliations:** 1 Assistant Professor of Preventive Medicine and Public Health and Preventive Medicine. School of Medicine. Avenida de la Investigación, 60, 18071, Granada, Spain; 2 PhD, Tenured Lecturer of Special Care in Dentistry and Gerodontology. School of Dentistry. University of Granada. C/ Campus Cartuja s/n, 18071, Granada, Spain; 3 PhD, Tenured Lecturer of Prosthodontics. School of Medicine. University of Salamanca. C/ Alfonso X El Sabio s/n. 37007, Salamanca, Spain; 4 PhD, Assistant Professor of Prosthetic Dentistry. School of Dentistry. University of Granada. C/ Campus Cartuja s/n, 18071, Granada, Spain; 5 PhD, Professor of Preventive and Community Dentistry. School of Dentistry. University of Granada. C/ Campus Cartuja s/n, 18071, Granada, Spain; 6 Instituto de Investigación Biosanitaria ibs.GRANADA, Complejo Hospitales Universitarios de Granada/Universidad de Granada, 18071 Granada, Spain

## Abstract

**Background:**

To improve eradication strategies of health-compromising behaviors between oral cancer survivors, this study aimed to explore the extent of clustering of risk behaviors and to assess possible factors associated.

**Material and Methods:**

A cross-sectional study was carried out among oral cancer patients at least 6 months after treatment. They completed a questionnaire about smoking, alcohol consumption, oral hygiene habits and dental visits. Presence of clusters was evaluated through pairwise Pearson correlations and principal component analysis. Factors associated with each identified cluster were analyzed with multivariate models.

**Results:**

Among 142 patients, 14.8% smoked, 51.7% consumed alcohol, 52.1% performed oral hygiene less than twice a day, and 74.6% visited to dentist when there was a problem or never. There were two distinct clusters: smoking-alcohol consumption (general risk behaviors cluster) and oral hygiene-dental attendance (oral risk behaviors cluster). Multivariate analysis showed significant associations between males and both clustering patterns of health compromising behaviors, patients with clinical stage I or with longer follow-up and the presence of general risk behaviors cluster and worse social class and the presence of oral risk behaviors cluster.

**Conclusions:**

A high proportion of patients treated for oral cancer presented health-compromising behaviors occurring in clusters which reinforce the need for health promotion strategies to target multiple behaviors. Factors analyzed suggest that chances of having detrimental behavioral clustering are higher in male, patients with clinical stage I, with lower social class and those with longer follow-up after treatment.

** Key words:**Oral cancer, health risk behaviors, survivors.

## Introduction

Cancers of head and neck area comprise malignancies of oral cavity, pharynx and larynx. Oral cancer is the most common between them with reports of 300373 new cases worldwide in 2012 (1). New advances in the modalities and applications of treatments have resulted in a better control of this cancer and in a decrease in short-time mortality (2). Nevertheless, the high propensity for multiple primary carcinomas and relapses in the oral cavity (3) remains being a challenge for professionals.

Lifestyle behaviors like smoking and alcohol consumption have been strongly related to oral cancer (4,5) and to higher incidence of second primary malignancies (6). More controversially, oral hygiene habits and dental visits are also associated variables with oral cancer appearance and survival (7,8). It has been demonstrated that a large proportion of oral cancers could be preventable by the eradication of these modifiable risk behaviors (9). With the demonstrated existence of a link between health-compromising behaviors and recurrence of oral cancer, it seems reasonable that surviving patients give up these risk habits. However, many patients diagnosed of oral cancer continue with these health-compromising behaviors (8,10).

It has been suggested that risk behaviors do not usually occur in isolation but instead concur as clusters or bundles (11). Therefore, changing two or more behaviors at the same time could have higher benefits than the focusing on only one (12). To carry out these behavior changes effectively, it is important to know which behaviors concur and which factors they share. These issues are not yet clear. Most of studies are focused on general health risk behaviours (13) so little is known about oral health related behaviours (11). Moreover, these studies focus mostly on the general population when clusters can differ between samples (13).

The aim of this study were to determine the extent of clustering of risk behaviors in oral cancer patients at least 6 months post-treatment and to explore possible factors associated with them.

## Material and Methods

-Patients

This study was carried out in the Department of Maxillofacial Surgery of Virgen de las Nieves University Hospital in Granada (Spain). Study participants were oral/oropharyngeal cancer patients who had been treated at least 6 months earlier to avoid that the acute phase of recovery and adaptation to the new situation could alter our results and with no recurrence of the disease. These criteria were fulfilled by 145 patients, of whom 142 (97.9%) gave their written informed consent and were included in the study, which was approved by the Ethics Committee of the University of Granada.

-Measurements

Patients were asked after a follow-up visit by their doctor. The interview was conducted by a single researcher. Four health-related behaviors related to oral cancer were assessed: tobacco smoking, alcohol consumption, oral hygiene, and frequency of visits to the dentist. Patients were classified as current smokers or non-smokers according to their response to the question: “Do you smoke cigarettes, pipe or cigars at all nowadays?”. Patients were classified as non-alcohol drinkers (never or hardly ever) or current alcohol drinkers (occasionally or more often) according to their response (never/hardly ever, occasionally, weekly, or daily) to the question: “How often do you drink alcohol nowadays?”. Oral hygiene of patients were categorized according to their response (twice or more a day, once a day, less than once a day) to the question “How often do you clean your teeth, dentures or gums nowadays?”. Patients were also divided between those cleaning twice or more a day and those cleaning less frequently. The frequency of visits to the dentist was recorded by asking patients whether they visited “regularly or occasionally” or “only when there is a problem or never”.

Information was also gathered on the sex, age, and social class of participants. Tumor site, clinical stage, length of follow-up, presence of comorbidities and type of treatment was collected from their medical records. An oral exploration was carried out to analyse the following variables: posterior tooth units (pairs of occluding natural, restored or fixed prosthetic postcanine teeth: molars=2 units; bicuspids=1 unit), clinical attachment loss (sum of probing death and distance between the cemento-enamel junction and the gingival margin) and unstimulated salivary flow.

-Statistical analysis

SPSS version 17.0 (IBM Inc., Chicago, IL) was used for the statistical analysis. A descriptive analysis was performed of the socio-demographic and clinical variables. Pairwise Pearson correlations and principal component analysis with Varimax rotation were performed to identify co-occurrence of behaviors.13 Logistic regression models were run on the association of identified clusters (dependent variable) with age, sex, social class, clinical stage, follow-up, tumor site and type of treatment. These independent variables were recoded to create more balanced groups when were analyzed. All variables were simultaneously entered into the model to adjust for possible associations. Strong correlations were found between clinical stage and type of treatment to be introduced both in the models. Clinical stage was selected for inclusion based on the literature (14). The level of significance established was *p* < 0.05.

## Results

 Table 1 describes the socio-economic and clinical characteristics of the participants. The mean age was 65.2 (standard deviation: 12.9) years and 64.1% were male. More than half of the patients were from the lowest social class (V). Most frequent oral cancer localization was the tongue, and clinical stages I and IV were the most prevalent. The mean follow-up from treatment was 4.9 (4.3) years and the most common treatment was surgery alone (52.1%) followed by surgery plus radiotherapy (30.3%).

**Table 1:**
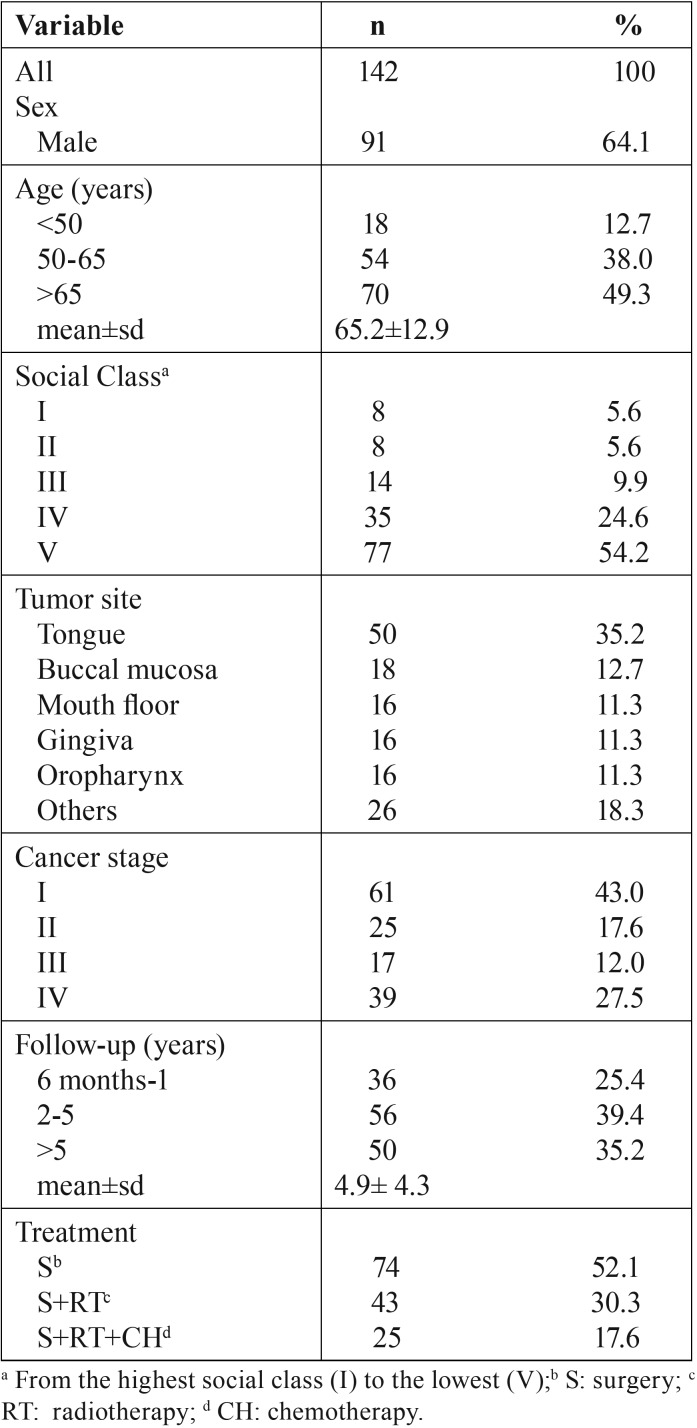
Description of socio-demographic and clinical data variables of patients treated for oral cancer (n=142).

Current smoking habit was reported by 14.8% of patients, alcohol was consumed weekly or daily by 51.7%, oral hygiene was performed less than twice a day by 52.1% and the dentist was never visited or only when there was a problem by 74.6% of patients. Significant correlations were found between smoking and alcohol, between smoking and poor oral hygiene (less than twice a day) and between dental visits and poor oral hygiene; these correlations were moderate or weak with coefficients ranging from 0.235 to 0.446 (data no shown).

 Table 2 shows the results of factor analysis. A two-factor solution was found, explaining about 71% of the variance in the data. Smoking and alcohol consumption were grouped into one factor (general risk behaviors cluster) and oral hygiene (less than twice a day) and dental attendance (only when there was a problem or never) in another factor (oral risk behaviors cluster). The Kaiser-Meyer-Olkin measurement was acceptable (0.522) and Bartell’s test sphericity was significant (<0.001). These considerations allowed us to conduct factor analysis legitimately.

**Table 2:**
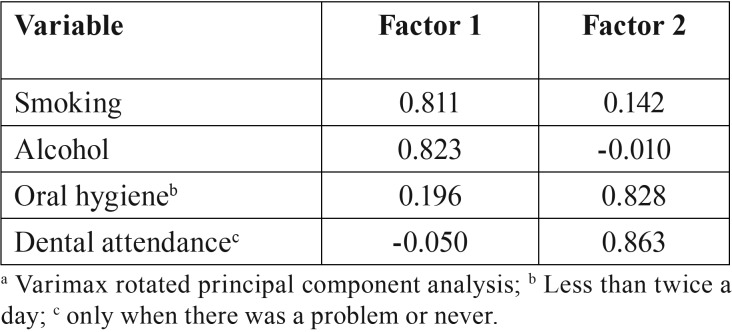
Description of results of principal component analysis in patients treated for oral cancer (n=142).

Comparison of oral status variables and disease status between presence and absence of identified clusters is presented in the  Table 3. Patients with presence of oral risk behaviors cluster had significantly lower mean of unstimulated saliva flow (*p*= 0.023) and functional posterior tooth units (*p*< 0.001) and higher clinical attachment loss (*p*< 0.001) compared to patients without this cluster.

**Table 3:**
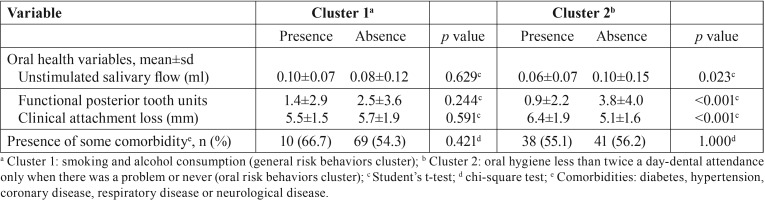
Comparison of oral health variables and presence of comorbidities between patients with general risk behaviors cluster (cluster 1) and oral risk behaviors cluster (cluster 2).

 Table 4 shows the association between identified clusters and patient characteristics. Men were significantly more likely to present risk behavior clusters (OR: 5.81; 95% CI: 1.09-31.04 for general risk behaviors cluster and OR: 7.96; 95% CI: 3.22-19.71 for oral risk behaviors cluster). Patients with clinical stage I and patients with longer follow-up were significantly more likely to present general risk behaviors cluster (OR: 7.35 and 7.73 respectively). Presence of oral risk behaviors cluster was also significantly associated with worse social class (OR: 3.94; 95% CI: 1.70-9.16).

**Table 4:**
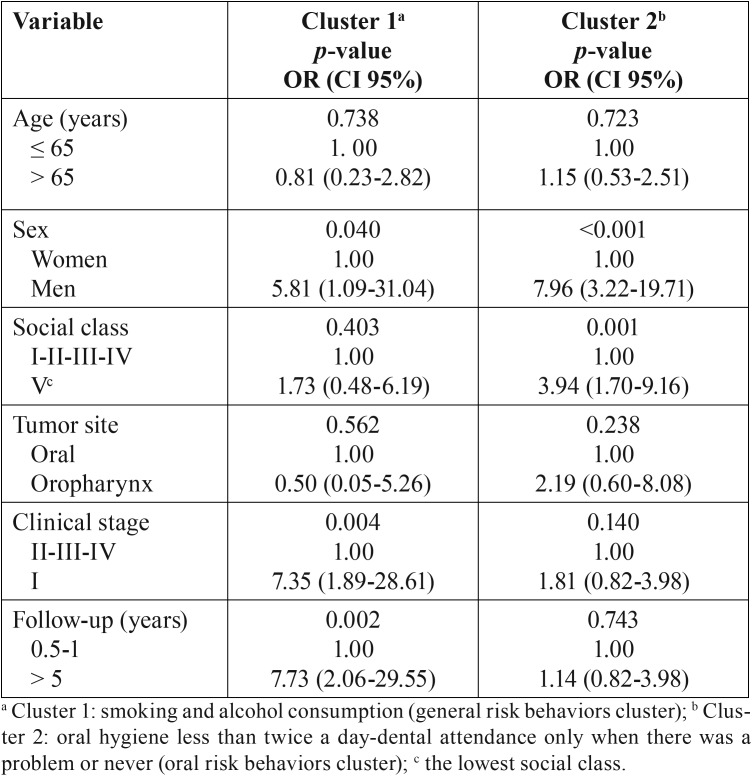
Multivariate logistic regression with risk behavior clusters as dependent variables in patients treated for oral cancer (n=142).

## Discussion

This study reveals the persistence of modifiable risk factors in a substantial proportion of patients who have been treated for oral cancer. Identified risk behaviors clusters (smoking-alcohol consumption and oral hygiene-dental attendance) were associated with sex, social class, clinical stage, and follow-up of patients.

There are some potential limitations in this study. Data on health-compromising behaviors were based on self-reports, which may produce an underestimation of their prevalence. The cross-sectional design was a further study limitation, preventing analysis of these behaviors over time. Nevertheless, the results of this study have allowed to draw conclusions and achieve the objectives previously proposed with the use of statistical analysis (clustering analysis) which offer high advantages to this approach (15).

In our study, current smoking was reported by 14.8% of patients. Published rates of smoking in patients treated for oral cancer widely range between 20.0% and 56.0% (16-20). These differences may be attributable to differences among studies in the post-treatment time interval. Consistently with previous studies (19,21) patients were more likely to continue with alcohol consumption (weekly or daily alcohol consumption was reported by 51.7% of patients) than smoking. This result reveals a possible educational deficiency in patients treated for oral cancer: they could have greater awareness of tobacco as a risk factor in comparison with alcohol. In fact, one of the most important information needs among oral cancer survivors is how to live a healthy lifestyle after treatment (22). Both behaviors are related as cluster (general risk behaviors cluster) suggesting a joint approach. Males were significantly more likely to have this cluster with high odds ratio (OR: 5.8; 95% CI: 1.09-31.04) and patients with earlier clinical stage and longer follow-up time were more likely to present this cluster as observed in other studies analyzing these behaviors independently (17,19). It may be attributable to the lesser awareness of the severity of their disease, the receipt of less radical treatment, the greater expectation of survival, and/or less concern about the possibility of recurrence over time. Taking into account these results and the evidenced efficacy and cost-effectiveness of tobacco treatment into cancer care (23), preventive health education integrating the alcohol issue should be continued over time in oral cancer patients.

Poor oral hygiene is known to be associated with oral cancer onset (16). More than half of patients cleaned their mouth less than twice a day. This oral hygiene pattern can be explained because aftermath of oral cancer treatments can sometimes hinder oral hygiene practices (24). Moreover, edentulous patients (25.4 % of our sample) may have an inadequate appreciation of the need of oral hygiene for not presenting teeth in the mouth. Around three-quarters of the patients only visited their dentist when there was a problem (if at all), similar to the findings of other studies in oral cancer patients (25,26). The fact that oral cancer patients are typically followed up at their hospital center or a low awareness about the role of the dentist in oral cancer process (27) could explain the low frequency of visits to dentist. Both behaviors: oral hygiene (less than twice a day) and dental attendance (only when there was a problem or never) were also identified as cluster (oral risk behaviors cluster) and were significantly associated with worse oral health status. Again, males were significantly more likely to have these risk behaviors (OR: 7.96; 95% CI: 3.22-19.71) and, in line with findings in healthy elderly (28), we found a relationship observed between the lowest social class and oral risk behaviors cluster. These finding shows the desirability of a multidisciplinary team for a better approach of these patients along time with the inclusion of the dentists. Dentists could instruct patients the different oral hygiene techniques available (29) with special attention to males and patient with lowest social class.

To our best knowledge, this is the first study to analyze the clustering of risk behaviors in oral cancer patients. Knowledge about clustering of risk behaviors may help intervention strategies to focus on behaviors sharing the same underlying source. This way, if an intervention succeeds in changing a particular behavior related behaviors may also change. According our findings, long-term supportive interventions are necessary after oral cancer treatment to inform to patients about risk behaviors and about techniques for their minimization. Coordinated planning among health professionals involved is required to establish an oral and general health preventive programs at the diagnosis and during the follow-up. The factors evaluated (sex, social class, clinical stage and follow-up of patients) allow that these programs can be more effectively targeted.

In conclusion, health-compromising behaviors persist in a substantial proportion of oral cancer patients at 6 months post-treatment occurring in clusters: smoking-alcohol consumption and oral hygiene-dental attendance. Male gender, lower social class, patients with clinical stage I and those with longer follow-up were more likely to have clustering of risk behaviors and special attention should be paid to these subgroups. It would be interesting to continue with the follow-up of this patient cohort to evaluate the possible implications of our results in outcomes as recurrence rate or survival. More studies are needed for further research on risk behaviors clusters and their factors associated in oral cancer patients.
